# *VEGF-A* Gene Expression Profiling in Northern Thai Gastric Cancer Patients and Potential for Anti-VEGF Therapy

**DOI:** 10.3390/diagnostics16182980

**Published:** 2026-09-15

**Authors:** Amonnat Sukhamwang, Hathairat Thananchai, Peticha Tanprasert, Pornngarm Dejkriengkraikul, Supachai Yodkeeree, Sirikan Limpakan (Yamada)

**Affiliations:** 1 Department of Biochemistry, Faculty of Medicine, Chiang Mai University, Chiang Mai 50200, Thailand; amonnat_s@cmu.ac.th (A.S.); pornngarm.d@cmu.ac.th (P.D.); supachai.y@cmu.ac.th (S.Y.); 2 Department of Microbiology, Faculty of Medicine, Chiang Mai University, Chiang Mai 50200, Thailand; hathairat.t@cmu.ac.th; 3 Division of Upper Gastrointestinal Surgery and Endoscopy, Surgery, Faculty of Medicine, Chiang Mai University, Chiang Mai 50200, Thailand; peticha.t@cmu.ac.th

**Keywords:** *VEGF-A* gene expression, *IL-8* gene expression, gastric cancer, Northern Thai gastric cancer profiling, chemotherapy

## Abstract

**Background/Objectives**: Gastric cancer (GC) remains a major burden in Northern Thailand. Elevated interleukin-8 (*IL-8*) and vascular endothelial growth factor A (*VEGF-A*) expression are linked with poor prognosis and tumor progression in gastric cancer. However, the concordance of *VEGF-A* and *IL-8* gene expression profiles and their association with chemotherapy and survival outcomes remain unclear. This study aims to characterize *VEGF-A* and *IL-8* gene expression profiles to address these knowledge gaps. **Methods**: We analyzed mRNA expression of *VEGF-A* and *IL-8* in 83 gastric mucosal tissue samples from advanced GC patients using RT-qPCR. Expression profiles were reported in distribution format, Kaplan–Meier survival curves, and multivariable Cox models to identify independent prognostic factors. Pre- and post-treatment expression patterns were observed. **Results**: *IL-8* overexpression was found in 83.33% of patients, while *VEGF-A* overexpression was found in 44.23%. Baseline *IL-8* and *VEGF-A* expression levels were not significantly associated with overall survival. However, paired sample analysis showed post-chemotherapy suppression of *IL-8* and *VEGF-A* expression, with a small percentage of downregulation in a subgroup of patients 33.3% and 30%, respectively. **Conclusions**: *VEGF-A* and *IL-8* display concordant distribution of expression patterns but differ in downregulation by the same chemotherapy regimen. Although baseline expression lacked independent prognostic value, non-frequent downregulation of *VEGF-A* and *IL-8* occurred post-chemotherapy in Thai advanced gastric cancer patients, suggesting its potential to be a biomarker for chemoresistance. These findings support personalized molecular profiling of *VEGF-A* expression to identify candidates who may get the benefit from early VEGF-targeted therapies in Thai GC patients.

## 1. Introduction

Gastric cancer (GC) remains a formidable global health burden. According to GLOBOCAN, GC is the fifth most frequently diagnosed malignancy and the fourth leading cause of cancer-related death worldwide [1]. Despite declining incidence in certain Western nations, prevalence remains high across East and Southeast Asia, including China, Japan, South Korea, and Thailand [2,3]. Within Thailand, GC continues to pose significant challenges, particularly in the northern region, where socioeconomic disparities, dietary habits, environmental exposures, and the prevalence of Helicobacter pylori infection may contribute to unique epidemiological and molecular profiles [4,5,6,7]. Although national cancer screening initiatives have improved early detection for several cancers, GC in Thailand is still often diagnosed at advanced stages when curative strategies are limited. Indeed, the five-year survival rate for advanced GC remains under 30%, underscoring the urgent need for more effective therapeutic approaches and reliable predictive biomarkers [8,9].

Current standard of care for GC involves a multimodal approach, combining surgical resection when feasible with neoadjuvant or adjuvant chemotherapy and radiotherapy. In patients with unresectable or metastatic disease, systemic chemotherapy remains the mainstay of therapy, although resistance inevitably develops and median survival remains poor. In recent years, molecular-targeted therapies and immunotherapy have emerged as promising adjuncts, with anti-angiogenic strategies drawing particular interest due to the central role of tumor vascularization in malignant growth and dissemination [10,11,12].

Angiogenesis—the formation of new blood vessels from existing vasculature—is widely recognized as a hallmark of cancer [13]. Tumors rely on neovascularization to secure oxygen, nutrients, and routes for metastasis [14]. Among the regulators of angiogenesis, the vascular endothelial growth factor (VEGF) family, and particularly VEGF-A, has been intensively studied and therapeutically targeted. VEGF-A binds to VEGFR-1 (Flt-1) and VEGFR-2 (KDR/Flk-1) on endothelial cells, launching downstream signaling cascades that support endothelial proliferation, migration, and survival. Overexpression of *VEGF-A* is common across many solid tumors, including gastric, colorectal, breast, and lung cancers; it is frequently associated with increased microvessel density, lymph node metastasis, and poorer survival outcomes [15,16]. In gastric cancer, elevated *VEGF-A* expression has been correlated with advanced disease stage and has led to its consideration as both a prognostic biomarker and a potential therapeutic target [17,18].

Multiple anti-VEGF agents have been evaluated in gastric cancer. Bevacizumab—a monoclonal antibody against VEGF-A—has shown efficacy in cancers such as colorectal and breast, but results in GC trials have been inconsistent [19]. In contrast, ramucirumab, a monoclonal antibody targeting VEGFR-2, has demonstrated more promising results [20]. In the REGARD and RAINBOW phase III trials, ramucirumab (alone or in combination with paclitaxel) conferred significant improvements in progression-free survival and overall survival in patients with advanced gastric or gastroesophageal junction adenocarcinoma who had failed first-line chemotherapy [21,22,23]. Other agents under investigation include VEGF-trap fusion proteins (e.g., aflibercept) and multi-kinase inhibitors such as lapatinib and regorafenib, though their benefits appear restricted to selected patient subsets [24,25]. The key challenge remains that not all patients respond to anti-angiogenic therapy, highlighting the critical need for biomarkers to stratify likely responders.

In Asian populations, several studies and meta-analyses have shown that *VEGF-A* overexpression is associated with worse prognostic features in GC. For example, a meta-analysis of Asian GC cohorts reported that *VEGF-A*-negative tumors had significantly poorer overall survival (relative risk ~2.45) compared to *VEGF-A*-positive counterparts [26]. However, data specific to Thai patients are scarce, and studies specifically addressing northern Thailand are almost nonexistent. This paucity is problematic because regional genetic, environmental, and lifestyle factors may influence the biology of angiogenic signaling and therapeutic responsiveness.

Beyond angiogenesis, mounting evidence implicates inflammation as a driver of gastric tumorigenesis and resistance to therapy. Interleukin-8 (IL-8, also known as CXCL8) is a pro-inflammatory chemokine secreted by tumor cells, macrophages, and stromal elements in response to stress, cytokine stimulation, and exposure to chemotherapy [27]. IL-8 activates its receptors CXCR1 and CXCR2, promoting cancer cell proliferation, survival, epithelial-to-mesenchymal transition (EMT), angiogenesis, and modulation of the immune microenvironment [28]. Elevated *IL-8* gene expression has been linked to deeper tumor invasion, lymph node metastasis, and poor survival in GC [29,30]. Preclinical studies further suggest that IL-8 may synergize with VEGF-A. However, IL-8 has been shown to induce VEGF-A production in endothelial cells, thereby promoting angiogenesis in a feed-forward manner [31]. Some clinical studies have demonstrated elevated VEGF-A and IL-8 in gastric cancers relative to adjacent non-neoplastic mucosa, although direct correlations between the two were not always consistent [15,32].

Notably, chemotherapy itself can sometimes induce *IL-8* expression in tumor cells, potentially creating a pro-inflammatory and pro-angiogenic microenvironment that promotes tumor regrowth, resistance, or metastasis [33]. Despite these insights, little is known about the concurrent expression dynamics of *VEGF-A* and *IL-8* in Thai GC patients, particularly in relation to chemotherapy and treatment outcome.

This study seeks to fill these knowledge gaps by characterizing the expression profiles of *VEGF-A* and *IL-8* in gastric cancer tissues from patients in Northern Thailand. We aim to compare mRNA expression levels of *VEGF-A* and *IL-8* between tumor tissues and matched normal tissues, evaluate how expression changes following chemotherapy, and examine co-expression patterns to explore the interplay of angiogenic and inflammatory pathways. By integrating molecular data with clinicopathological factors, we intend to develop a more nuanced understanding of the angiogenic–inflammatory landscape in Northern Thai gastric cancer patients. Our goal is to inform biomarker-guided strategies that may improve selection for anti-VEGF therapies and potentially support combined targeting of IL-8 pathways in this specific population.

## 2. Materials and Methods

### 2.1. Patients

A total of 83 patients diagnosed with advanced-stage gastric cancer were enrolled from Maharaj Nakorn Chiang Mai Hospital in Thailand between 2015 and 2020. Informed consent was obtained from all participants before sample collection. Tissue specimens were collected in accordance with ethical standards and approved by the Research Ethics Committee of the Faculty of Medicine, Chiang Mai University (Approval No. SUR-2558-03015). All the patients were observed until the completion of the study or their deaths. All specimens were processed within a day of sample collection. The clinical features of all patients, including age at diagnosis, gender, site of disease, surgery type, and histological type, were obtained from clinical and pathological reports. All patients were diagnosed according to the criteria of the World Health Organization (WHO) and the Lauren Classification.

### 2.2. Cell Culture, RNA Isolation, and RT-qPCR Analysis

Human gastric adenocarcinoma cells (AGS cells) were cultured to a density of 2 × 10^6^ to 4 × 10^6^ cells before harvesting. Total mRNA was isolated from both harvested AGS control cells and the patient fresh gastric tumor biopsy specimens (tumor and matched normal tissues collected during diagnostic chromoendoscopy). Histopathological status and tissue adequacy were routinely verified by a pathologist prior to RNA extraction using TRIzol™ Reagent (Invitrogen™, Thermo Fisher Scientific, Waltham, MA, USA) according to the manufacturer’s protocol. Fresh biopsy specimens were immediately preserved in TRIzol™ reagent at −20 °C prior to RNA extraction. RNA concentration and purity were verified using NanoDrop 2000/2000c Spectrophotometers (Thermo Fisher Scientific, Waltham, MA, USA).

The extracted RNA was reverse-transcribed into complementary DNA (cDNA) using the High-Capacity RNA-to-cDNA Kit (Applied Biosystems, Waltham, MA, USA) on a Mastercycler^®^ nexus gradient machine (Eppendorf, Hamburg, Germany) [34]. Quantitative real-time PCR (RT-qPCR) was performed using a qRT-PCR ABI™ 7500 Fast & 7500 Real-Time PCR system and analyzed via QuantStudio 6 Flex real-time PCR software v1.0 (Applied Biosystems, Waltham, MA, USA). Target gene expressions were assessed using specific Human TaqMan probe primers (Applied Biosystems, Waltham, MA, USA) for VEGF-A (Assay ID: Hs00900055_m1) and IL-8, with Glyceraldehyde-3-phosphate dehydrogenase (GAPDH; Assay ID: Hs02758991_g1) serving as the internal reference control matched to the template copy number. Relative gene expression analysis was performed using the 2^(−ΔΔCT)^ method, utilizing both raw relative quantitation (RQ) values and log_10_-transformed data to ensure normalization (Log_10_RQ). All expression values were normalized against the baseline levels of the AGS cell line controls.

For clinical interpretation, gene expression levels were categorized based on the definitive direction of the relative expression shift between the tumor tissue and its matched normal counterpart (Tumor/Normal ratio). Patients were then categorized into two distinct expression groups based on defined thresholds: ‘Normal expression’ was defined as a Log10 fold change < 1 (representing a less than 10-fold increase relative to its matched normal counterpart), whereas ‘Overexpression’ was defined as a Log10 fold change ≥ 1 (representing a 10-fold or greater increase relative to its matched normal counterpart).

### 2.3. Statistics

Statistical analysis was performed using Jamovi (Version 2.6), an open-source statistical software (The jamovi project, 2025, https://www.jamovi.org). For the clinical data, the endpoint was the overall survival (OS), which was calculated from the date of the first chemotherapy treatment to the date of death. Survival curves were plotted using the Kaplan–Meier method, and group differences in the survival curves were investigated by the log-rank test. Cox regression analysis was used for univariate and multivariate analyses. A Cox proportional-hazard model was used to identify variables that were independently associated with OS. Confounders (including age, sex, histological grade, therapy type) were selected for inclusion in multivariable Cox models based on their established clinical relevance in gastric cancer prognosis, rather than automated stepwise algorithms. Missing data were handled using a complete-case analysis approach, wherein only patients with complete records for all included covariates were analyzed in each respective model. A *p*-value < 0.05 was considered statistically significant.

## 3. Results

### 3.1. Basal Expression Level of IL-8 and VEGF-A in Northern Thai Gastric Cancer Cohort

**Figure 1:**
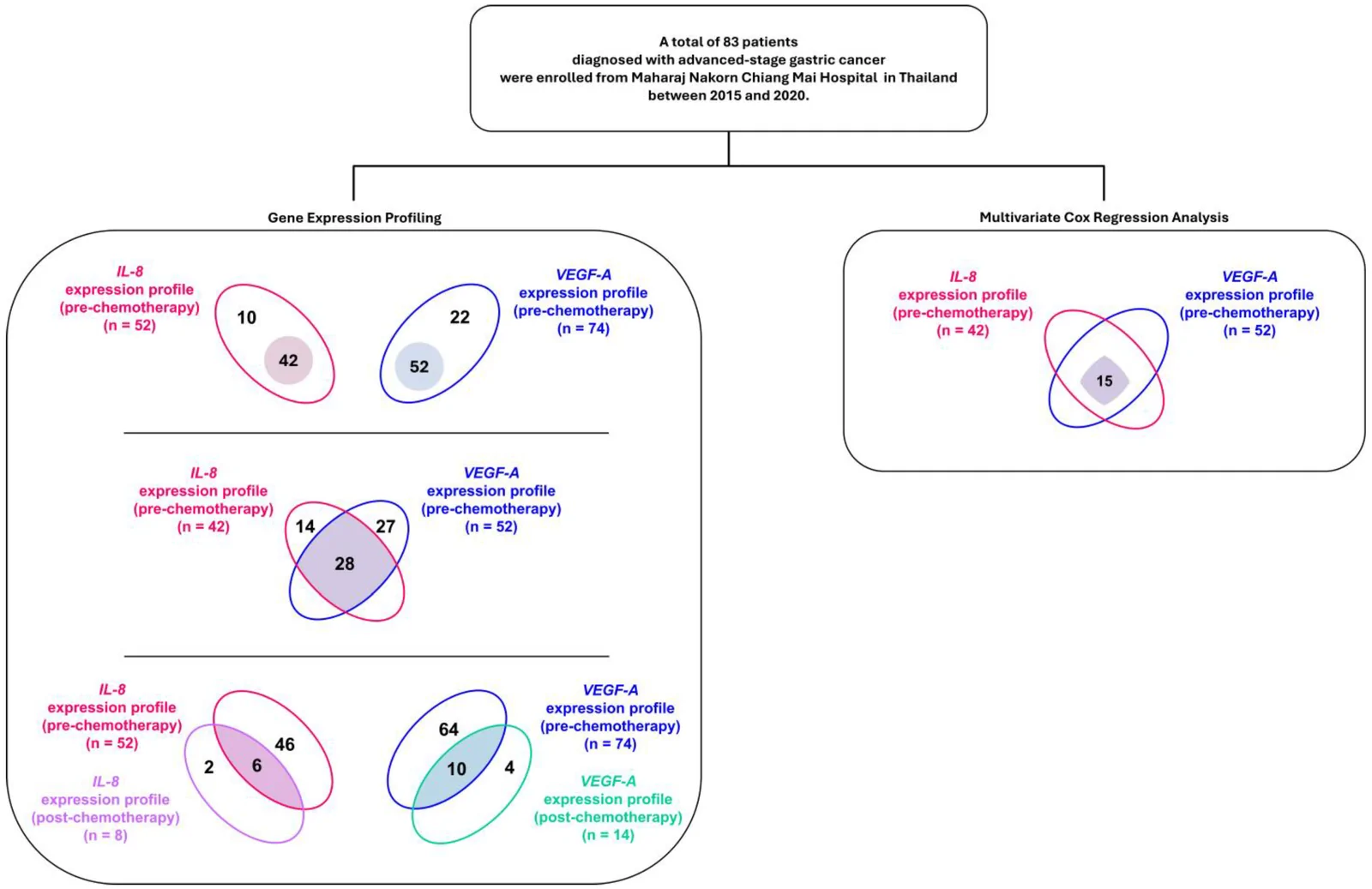
Flowchart of data availability and cohort assignment for downstream analyses.

To characterize the molecular landscape of angiogenic and inflammatory signaling in gastric cancer, we analyzed the expression profiles of vascular endothelial growth factor A (*VEGF-A*) and interleukin-8 (*IL-8*) in tissue samples obtained from Northern Thai gastric cancer patients. Samples were collected before and after chemotherapy. Quantitative gene expression analysis was conducted using the 2^−ΔΔCT^ method, with *GAPDH* serving as the internal control. All values were normalized to AGS gastric cancer cells to assess differential expression. Clinical characteristics of the cohort revealed that most patients presented with poorly differentiated tumors and were diagnosed at an advanced pathological stage, as summarized in Table 1. The distribution of data availability across the cohort and its subsequent stratification for downstream gene expression profiling and multivariable survival analyses are illustrated in Figure 1.

**Figure 2:**
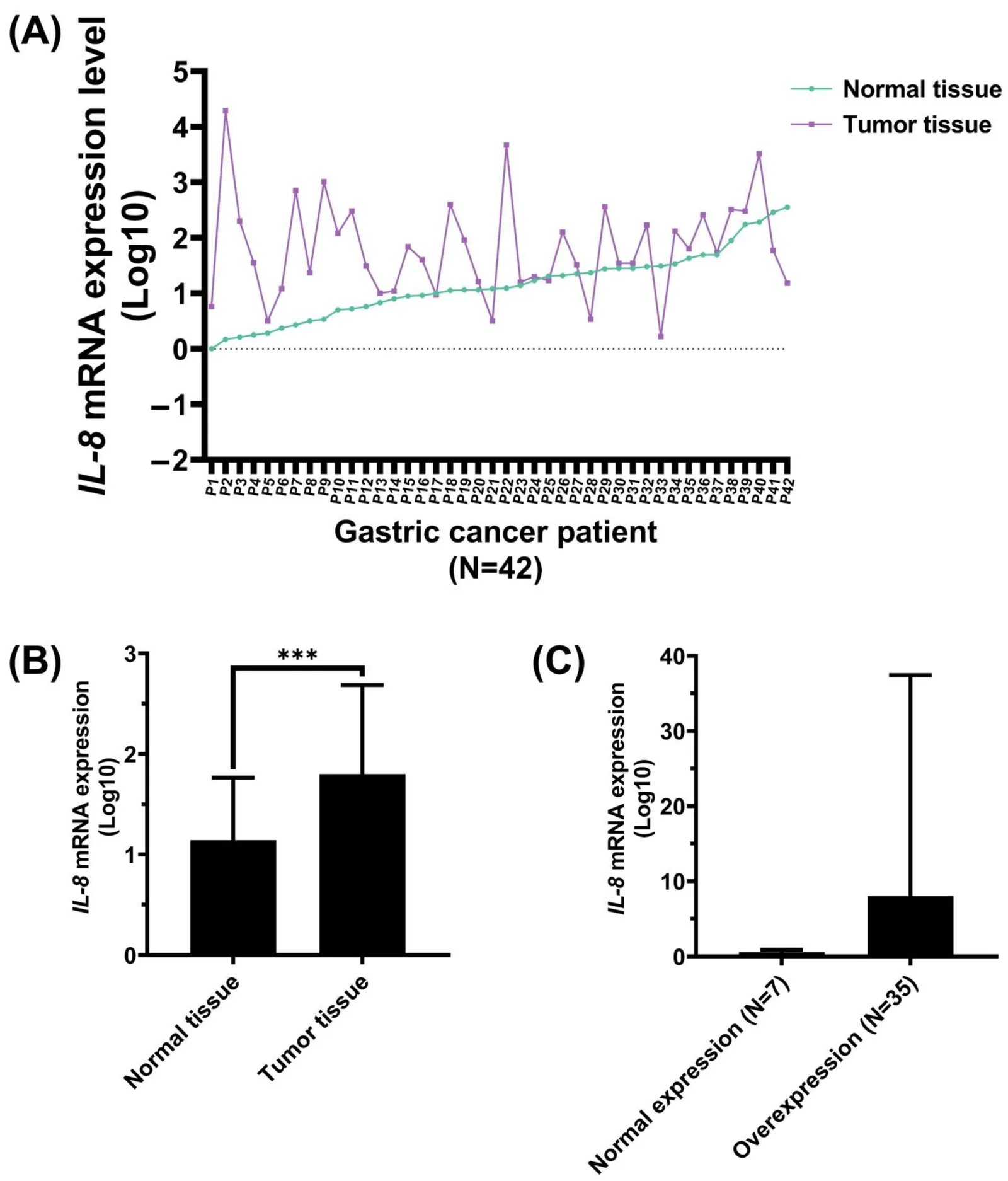
Analysis of *IL-8* mRNA expression levels in gastric cancer tissues. (**A**) Pairwise comparison of *IL-8* mRNA expression levels (Log_10_, relative to AGS reference cells) between paired normal and tumor tissues across individual patients (*n* = 42). The horizontal dotted line indicates the baseline expression level (Log_10_ fold change = 0). (**B**) Bar plot displaying the mean ± SD of Log_10_ *IL-8* mRNA expression levels in normal tissue versus tumor tissue (*n* = 42). (**C**) Bar plot displaying the mean ± SD of Log_10_ *IL-8* mRNA expression levels in tumor tissues, stratified into Normal expression (<1; *n* = 7) and Overexpression (≥1; *n* = 35) groups based on the tumor-to-normal expression shift relative to AGS cells. *** indicates *p*-value < 0.001.

**Figure 3:**
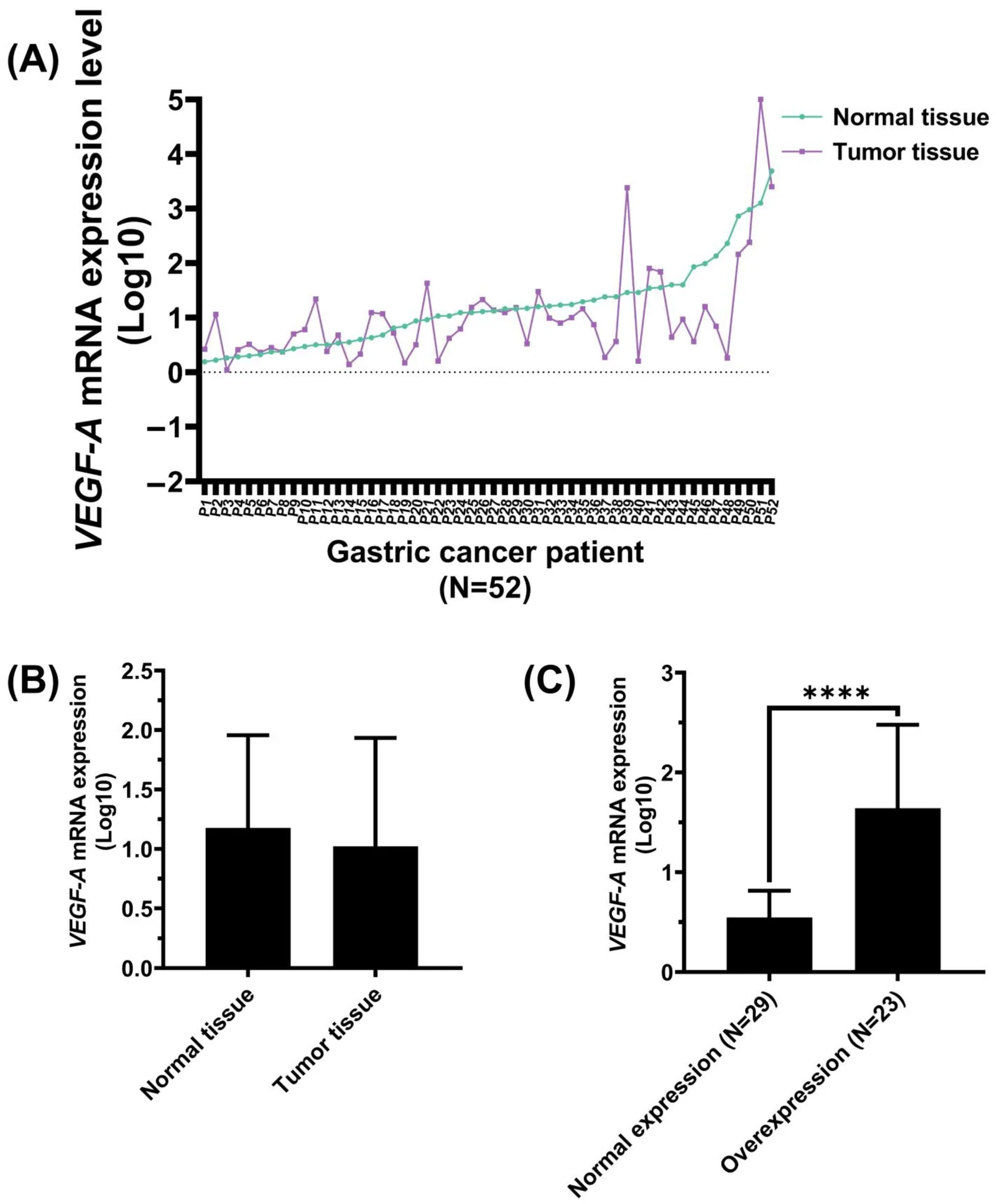
Analysis of *VEGF-A* mRNA expression levels in gastric cancer tissues. (**A**) Pairwise comparison of *VEGF-A* mRNA expression levels (Log_10_, relative to AGS reference cells) between paired normal and tumor tissues across individual patients (*n* = 52). The horizontal dotted line indicates the baseline expression level (Log_10_ fold change = 0). (**B**) Bar plot displaying the mean ± SD of Log_10_ *VEGF-A* mRNA expression levels in normal tissue versus tumor tissue (*n* = 52). (**C**) Bar plot displaying the mean ± SD of Log_10_ *VEGF-A* mRNA expression levels in tumor tissues, stratified into Normal expression (<1; *n* = 29) and Overexpression (≥1; *n* = 23) groups based on the tumor-to-normal expression shift relative to AGS cells. **** indicates *p*-value < 0.0001.

To ensure analytical rigor, specimens with non-detectable or baseline-equivalent expression levels (Log_10_RQ ≤ 0 relative to AGS reference cells in either tumor or paired normal sites) were excluded. Consequently, the final analysis cohort comprised *n* = 42 patients for *IL-8* (from an initial *n* = 52) and *n* = 52 patients for *VEGF-A* (from an initial *n* = 74). Pairwise analysis revealed heterogeneous expression patterns across individual patients for both target genes (Figure 2A and Figure 3A). For *IL-8*, overall mRNA expression was significantly elevated in tumor tissues compared to matched normal mucosal tissues (Figure 2B). Normal tissues exhibited a mean ± SD of 1.14 ± 0.62 (median = 1.02, range: 0.00 to 2.55), whereas tumor tissues showed a mean ± SD of 1.80 ± 0.89 (median = 1.62, range: 0.22 to 4.29). When stratified based on the tumor-to-normal expression shift relative to AGS cells, patients in the Normal expression group (<1; *n* = 7, 16.7%) demonstrated a mean ± SD of 0.58 ± 0.30 (median = 0.46, range: 0.15 to 0.97), while the Overexpression group (≥1; *n* = 35, 83.3%) exhibited a mean ± SD of 8.01 ± 29.44 (median = 1.66, range: 1.03 to 175.20) (Figure 2C). For *VEGF-A*, expression levels in normal tissues showed a mean ± SD of 1.18 ± 0.78 (median = 1.02, range: 0.19 to 3.69), while tumor tissues had a mean ± SD of 1.02 ± 0.91 (median = 0.89, range: 0.04 to 5.00) (Figure 3B). When stratified using the defined threshold, *n* = 29 (55.8%) patients were categorized into the Normal expression group with a mean ± SD of 0.54 ± 0.27 (median = 0.60, range: 0.11 to 0.94). In contrast, the Overexpression group (*n* = 23, 44.2%) showed a significantly higher mean ± SD of 1.64 ± 0.84 (median = 1.47, range: 1.00 to 4.91) (Figure 3C).

*IL-8* expression is a well-established poor prognostic marker in Thai gastric cancer [34,35] and has been implicated in promoting angiogenesis [36], epithelial-to-mesenchymal transition (EMT) [37], and resistance to chemotherapy [38,39]. Therefore, the high basal level of *IL-8* expression in this cohort may contribute to the observed aggressive tumor phenotypes. In summary, these findings suggest that IL-8 could serve as a more universal molecular driver of disease progression in Northern Thai gastric cancer patients, whereas VEGF-A may function as a stratification biomarker for targeted anti-angiogenic therapy in a specific patient subset.

### 3.2. Basal Co-Expression of IL-8 and VEGF-A in Northern Thai Gastric Cancer Cohort

To evaluate the combined expression patterns of *IL-8* and *VEGF-A*, an intersection analysis was performed using only patients with complete, paired data available for both target genes.

**Figure 4:**
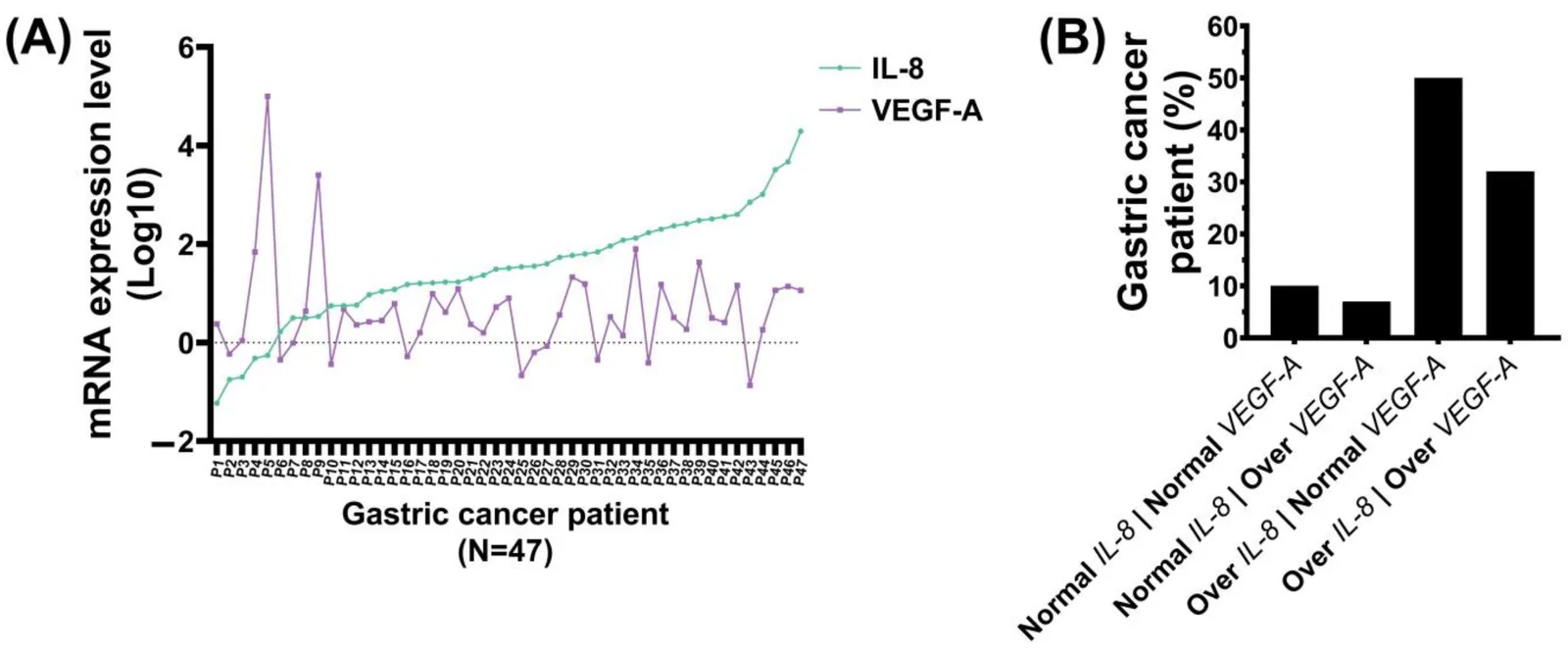
Co-expression distribution of *IL-8* and *VEGF-A* mRNA levels in pre-chemotherapy gastric cancer patients. (**A**) Line plot comparing the individual Log_10_ fold-change expression levels of *IL-8* (green) and *VEGF-A* (purple) across matched tumor site of patient specimens (*n* = 47). The horizontal dotted line indicates the baseline expression level (Log_10_ fold change = 0). (**B**) Bar graph illustrating the percentage distribution of gastric cancer patients (*n* = 28) stratified into four co-expression subgroups based on the relative tumor-to-normal mRNA expression shift in *IL-8* and *VEGF-A*: Normal *IL-8*|Normal *VEGF-A* (*n* = 3, 10.7%), Normal *IL-8*|Over *VEGF-A* (*n* = 2, 7.1%), Over *IL-8*|Normal *VEGF-A* (*n* = 14, 50.0%), and Over *IL-8*|Over *VEGF-A* (*n* = 9, 32.1%).

From the individual gene analysis cohorts (*n* = 42 for *IL-8* and *n* = 52 for *VEGF-A*), a total of *n* = 47 patients met the criteria for dual-gene evaluation at the tumor site (Figure 4A). Pairwise comparison of individual patient specimens demonstrated that baseline *IL-8* and *VEGF-A* expression generally followed a similar parallel trend in the majority of cases. However, *IL-8* expression levels were consistently higher than *VEGF-A* across most specimens, and a discordant expression pattern was observed in a specific subset of patients prior to treatment. Moreover, from the individual gene analysis cohorts (*n* = 42 for *IL-8* and *n* = 52 for *VEGF-A*), a total of *n* = 28 patients met the criteria for dual-gene evaluation at tumor/normal ratio. Based on the predefined expression thresholds (Log_10_ fold change ≥ 1 relative to matched normal tissue for overexpression), patients were categorized into four distinct co-expression subgroups (Figure 4B). The predominant pattern observed was the overexpression of *IL-8* with normal *VEGF-A* expression (Over *IL-8*|Normal *VEGF-A*), accounting for 50.0% of the cohort (*n* = 14/28). Simultaneous overexpression of both markers (Over *IL-8*|Over *VEGF-A*) was detected in 32.1% of patients (*n* = 9/28). In contrast, subgroups displaying normal *IL-8* expression represented minor proportions of the cohort, with normal *IL-8* expression with normal *VEGF-A* expression (Normal *IL-8*|Normal *VEGF-A*) and normal *IL-8* expression with overexpression of *VEGF-A* (Normal *IL-8*|Over *VEGF-A*) comprising 10.7% (*n* = 3/28) and 7.1% (*n* = 2/28) of patients, respectively. Overall, elevated *IL-8* expression—either alone (*n* = 14/28) or in combination with *VEGF-A* overexpression (*n* = 9/28)—was identified in 82.1% (*n* = 23/28) of the evaluated gastric cancer cohort. Furthermore, the observed heterogeneity in *VEGF-A* and *IL-8* co-expression emphasizes the complexity of the tumor microenvironment in gastric cancer, supporting the necessity of personalized molecular profiling to guide treatment strategies in this population.

### 3.3. Cox Regression Analysis and Survival Rate of VEGF-A Expression in Pre-Chemotherapy Treatment

To evaluate the independent prognostic value of clinical parameters alongside *IL-8* and *VEGF-A* expression profiles, Cox proportional hazards regression analysis was performed (Table 2). Due to the requirement for complete clinical, histological, treatment, and follow-up data across all evaluated parameters, the analytical subset was refined to *n* = 15 patients from the initial expression cohorts (*n* = 42 for *IL-8* and *n* = 52 for *VEGF-A*). In the multivariable Cox model, age ≥ 55 years was identified as an independent factor associated with increased mortality risk (hazard ratio [HR] = 8.97, 95% confidence interval [CI]: 1.07–75.3, *p* = 0.043). Conversely, adjuvant therapy showed a protective association compared to neoadjuvant therapy (HR = 0.10, 95% CI: 0.01–0.77, *p* = 0.027). Furthermore, VEGF-A overexpression demonstrated a statistically significant reduced risk in the multivariable model (HR = 0.19, 95% CI: 0.04–0.97, *p* = 0.046).

**Figure 5:**
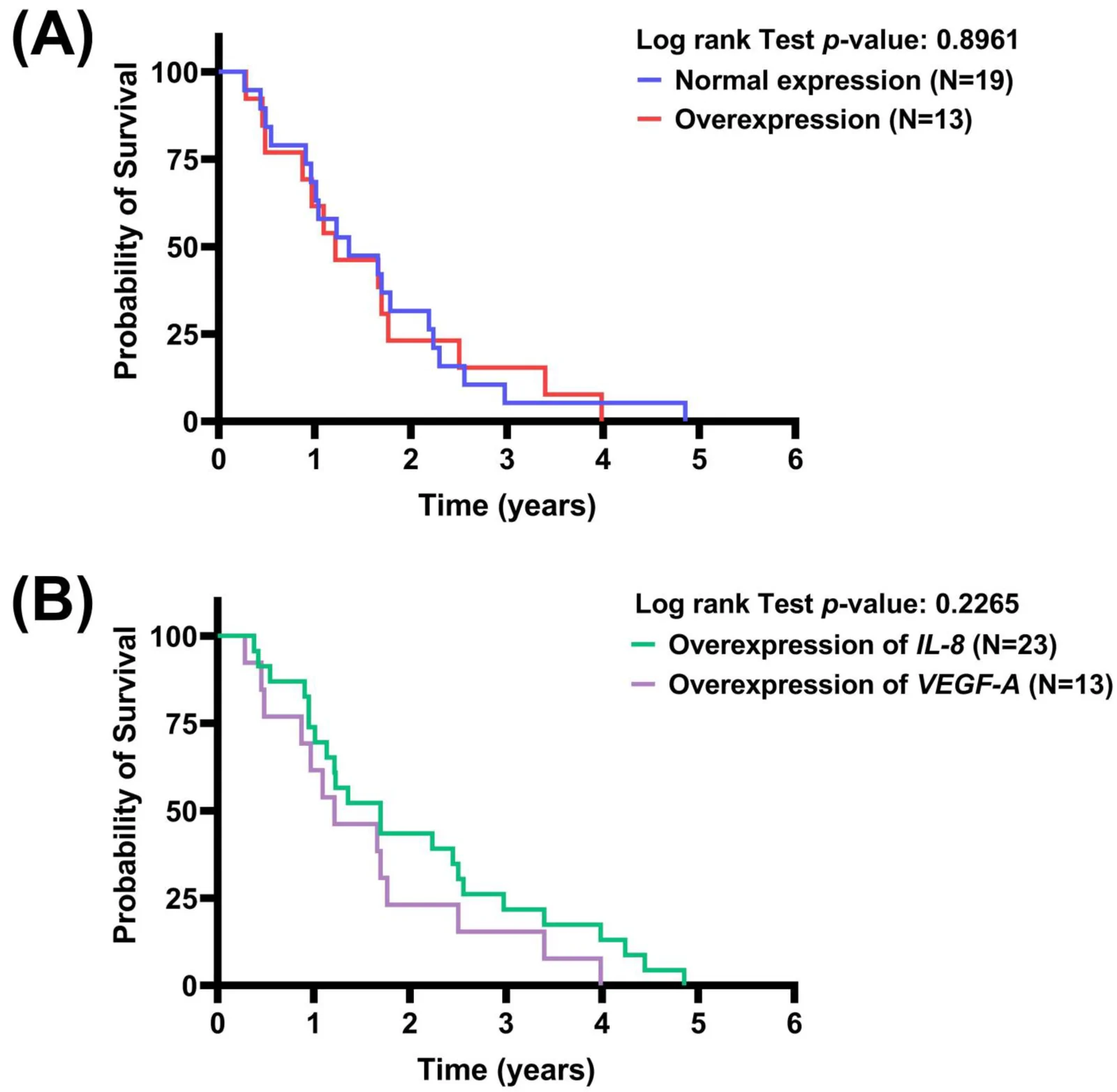
Kaplan–Meier overall survival curves comparing patient outcomes based on *IL-8* and *VEGF-A* overexpression. (**A**) Overall survival of gastric cancer patients with available survival data, stratified by normal expression (Log_10_ fold change < 1 relative to matched normal tissue; *n* = 19) versus overexpression (Log_10_ fold change ≥ 1 relative to matched normal tissue; *n* = 23) of *VEGF-A* (Log-rank test, *p* = 0.8961). (**B**) Overall survival of gastric cancer patients with available survival data, stratified by overexpression (Log_10_ fold change ≥ 1 relative to matched normal tissue) of *IL-8* (*n* = 23) versus *VEGF-A* (*n* = 13) (Log-rank test, *p* = 0.2265). Due to the lack of available normal expression samples for *IL-8*, the Kaplan–Meier plot comparing normal expression versus overexpression of *IL-8* is not presented.

To evaluate the clinical prognostic value of *VEGF-A* and *IL-8* expression, Kaplan–Meier survival analyses were performed on patients with available survival outcomes (Figure 5). First, overall survival was stratified by VEGF-A status, comparing cases with normal expression (*n* = 19) to those with overexpression (*n* = 13). No statistically significant difference in overall survival trajectory was observed between the two groups (Figure 5A; Log-rank test, *p* = 0.8961). Furthermore, comparing cumulative overall survival between the *IL-8* overexpression group (*n* = 23) and the *VEGF-A* overexpression group (*n* = 13) revealed no statistically significant difference in overall survival trajectory across the entire cohort (Figure 5B; Log-rank test, *p* = 0.2265). Specifically, patients with IL-8 overexpression exhibited a median survival of 1.70 years (20.3 months), compared to 1.22 years (14.6 months) in those with *VEGF-A* overexpression.

### 3.4. Heterogeneous Response of IL-8 and VEGF-A Expression to Chemotherapy

**Figure 6:**
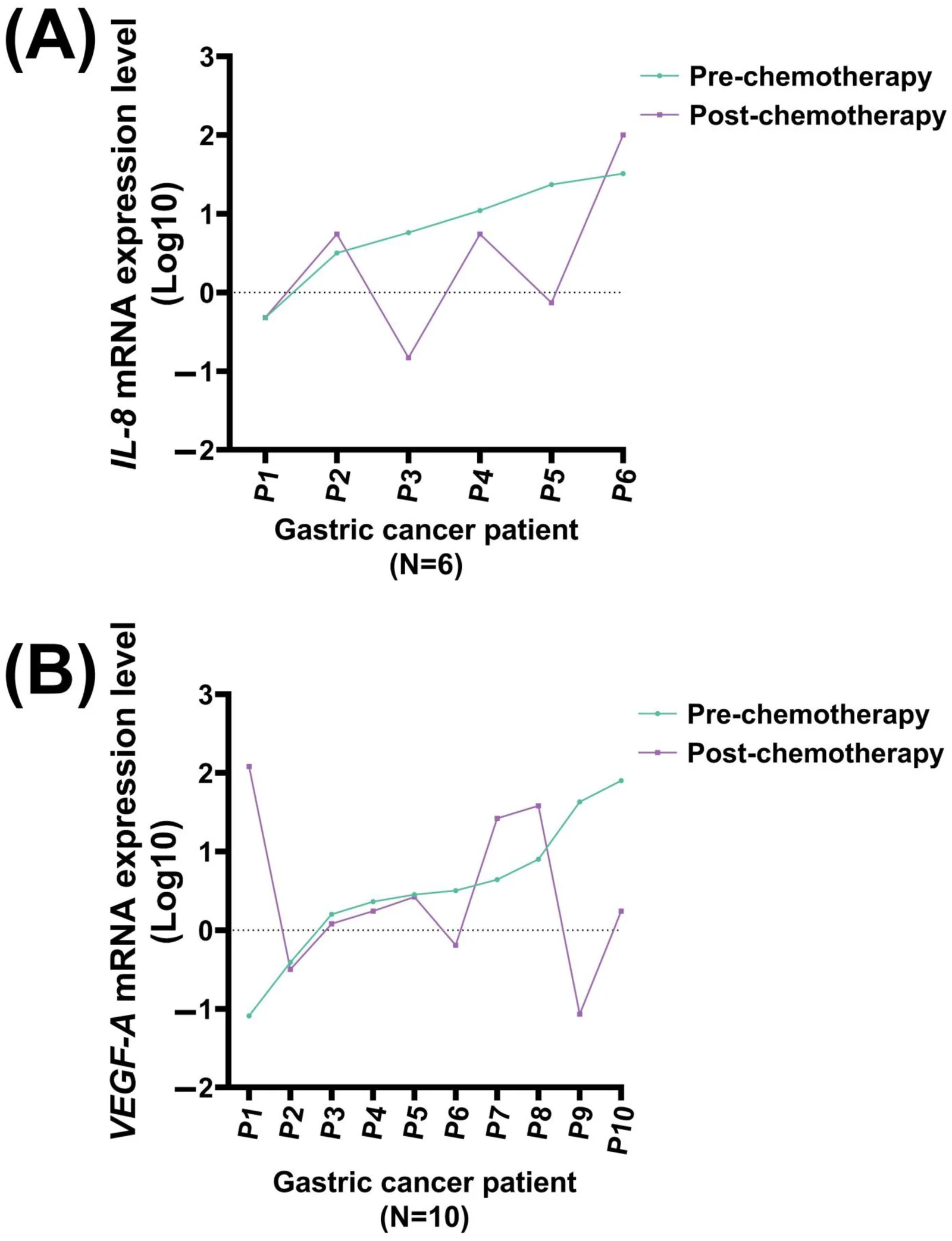
The expressions of (**A**) *IL-8* (*n* = 6) and (**B**) *VEGF-A* (*n* = 10) were compared between pre- and post- chemotherapy conditions. The horizontal dotted line indicates the baseline expression level (Log_10_ fold change = 0).

To investigate the effect of chemotherapy on *IL-8* and *VEGF-A* expression, we analyzed a subset of gastric cancer patients with paired specimens before and after chemotherapy. Matched expression profiles were available for *IL-8* (*n* = 6; Figure 6A) and *VEGF-A* (*n* = 10; Figure 6B). Pairwise evaluation revealed that chemotherapy suppressed *IL-8* expression in 33.33% of patients (2/6; P3 and P5), with levels noticeably declining compared to their pre-treatment baseline (Figure 6A). In contrast, evaluation of *VEGF-A* expression demonstrated patient-specific responses. Post-chemotherapy *VEGF-A* suppression was observed in 30% of patients (3/10; P6, P9, and P10), with a marked reduction observed only in P9; a pronounced and substantial reduction was clearly evident in only a single patient (1/10; P9), whereas P6 and P10 displayed minimal expression decreases (Figure 6B).

These observations suggest that, in certain cases, *VEGF-A* expression may be induced or restored during or following chemotherapy, potentially reactivating angiogenic signaling pathways. Altogether, these findings highlight the dynamic nature of molecular responses to chemotherapy and underscore the importance of monitoring *VEGF-A* expression throughout the treatment course. Although limited by the small sample size, the post-chemotherapy upregulation of *VEGF-A* in specific cases suggests that anti-angiogenic co-treatment strategies warrant further exploration. Such patients might benefit from combination regimens incorporating targeted anti-VEGF therapies alongside standard chemotherapy to suppress tumor revascularization and mitigate therapeutic resistance following first-line treatment.

## 4. Discussion

Gastric cancer remains a major global health challenge, ranking as the fifth most common malignancy and the fourth leading cause of cancer-related mortality worldwide, with more than one million new cases annually [40]. In endemic regions such as Northern Thailand, diagnosis often occurs at advanced stages, and the lack of robust prognostic biomarkers hampers timely clinical decision-making. Angiogenesis and inflammation are two central processes in gastric tumorigenesis, and vascular endothelial growth factor A (VEGF-A) and interleukin-8 (IL-8) are among the most extensively studied mediators of these pathways [41,42,43].

Our findings demonstrated that *VEGF-A* was upregulated in less than 30% of gastric cancer patients in the pre-chemotherapy setting (evaluated relative to the AGS reference control) in the pre-chemotherapy setting, reflecting considerable inter-patient heterogeneity. This aligns with previous studies showing that *VEGF-A* overexpression is present in approximately 30–50% of gastric cancer cases and is strongly correlated with advanced disease stage, lymph node involvement, and reduced survival [15,44]. In contrast, *IL-8* expression was elevated in over 70% of cases, consistent with its well-established role as a pro-inflammatory cytokine and promoter of angiogenesis, invasion, and metastasis [27,36,45]. Notably, the expression patterns of *VEGF-A* and *IL-8* were not correlated, suggesting independent regulatory mechanisms within gastric tumors. This reinforces the concept of molecular heterogeneity in gastric cancer and highlights the need for individualized biomarker profiling.

In terms of therapeutic implications, our longitudinal paired analysis revealed distinct post-chemotherapy dynamics between angiogenic and inflammatory signaling. Chemotherapy failed to suppress tissue *VEGF-A* expression in 30% of patients (3/10; P1, P7, and P8), with post-treatment samples demonstrating paradoxical upregulation. This rebound effect likely reflects an adaptive angiogenic escape mechanism [46]—a compensatory host response to cytotoxic stress previously described in advanced malignancies including esophageal squamous cell carcinoma [47]. Conversely, post-chemotherapy *VEGF-A* suppression was observed in 30.0% of patients (3/10; P6, P9, and P10), with a pronounced and substantial reduction clearly evident in only a single patient (1/10; P9). In contrast, chemotherapy suppressed *IL-8* expression in 33.33% of patients (2/6; *n* = 6), which closely aligns with the percentage of *IL-8* expression associated with chemoresistance reported previously in primary gastric cancer cells from the Thai population [48]. This differential response indicates that conventional chemotherapy reliably attenuates the tumor-associated inflammatory microenvironment, that angiogenic pathway reactivation occurred in a key subset of patients, and supports the rationale for combining chemotherapy with anti-VEGF targeted agents.

When evaluating prognostic implications, univariable analysis revealed no statistically significant association between overall survival and baseline expression of *IL-8* (*p* > 0.99) or *VEGF-A* (*p* = 0.51). In multivariable Cox regression analysis, *VEGF-A* overexpression yielded an adjusted HR of 0.19 (95% CI: 0.04–0.97, *p* = 0.046). However, this counter-intuitive protective trend should be interpreted with caution. Given the small sample size available for full multivariable modeling (*n* = 15), this statistical finding is highly likely influenced by limited sample power and potential model overfitting rather than a true biological protective effect of *VEGF-A*.

Furthermore, because all patients in this cohort received standard cytotoxic chemotherapy without targeted anti-angiogenic agents, therapeutic outcomes were predominantly driven by the backbone chemotherapy regimen. These constraints underscore that tissue *VEGF-A* and *IL-8* levels serve as complex biological markers of the tumor microenvironment rather than simple standalone prognostic indicators, reinforcing the need for validation in larger, prospectively stratified cohorts.

Crucially, these findings highlight the necessity of profiling baseline and longitudinal *VEGF-A* expression to guide personalized treatment strategies. Therefore, the baseline *VEGF-A* overexpression observed in 44.23% of patients and non-selected administration of VEGF-targeted therapy across all patients may lack clinical efficacy and are unlikely to yield clinical benefits. Assessing *VEGF-A* levels before chemotherapy, alongside monitoring post-induction levels—due to pronounced *VEGF-A* suppression occurring in only 10% of patients following chemotherapy—provides a potential biological framework to identify candidates who are more likely to benefit from anti-VEGF agents at the appropriate therapeutic window.

Consistent with our survival analyses, multivariable Cox regression indicated that baseline *IL-8* and *VEGF-A* expression profiles were not independent prognostic factors for overall survival. When directly comparing cumulative survival between patients harboring target gene overexpression, those with *IL-8* overexpression demonstrated a numerically longer median survival compared to those with *VEGF-A* overexpression. However, this difference did not reach statistical significance. Although previous global cohorts (e.g., TCGA) [29] and regional studies [38,48] have frequently linked *IL-8* upregulation to aggressive tumor behavior and adverse survival outcomes, the large-scale studies utilizing the TCGA dataset lack detailed of chemotherapy treatment profiles. Our findings suggested that *VEGF-A* and *IL-8* expression do not function as standalone prognostic predictors for standard chemotherapy, but rather serve as predictive stratification biomarkers for further precision-targeted therapies. The lack of statistical significance likely reflects the modest sample size and potential clinical heterogeneity within the subgroup, underscoring that tissue *VEGF-A* and *IL-8* may act as broader markers of angiogenic and inflammatory microenvironments, rather than independent drivers of survival outcomes in advanced gastric cancer.

Co-expression patterns of *IL-8* and *VEGF-A* varied significantly among individual patients. While some showed parallel expression trends (32.14% combined overexpression and 10.71% dual normal expression), others demonstrated marked divergence, dominated by isolated *IL-8* overexpression (50.00%). This distinct distribution suggests that these molecules are regulated by partially independent biological pathways within gastric tumors. While *IL-8* reflects a broader inflammatory microenvironment response, *VEGF-A* acts as a specific mediator of tumor vascularization. *IL-8* may act as a general marker of tumor-promoting inflammation, while *VEGF-A* functions as a specific driver of angiogenesis in a subset of patients. These findings support the rationale for combinatorial therapeutic strategies—such as anti-VEGF agents alongside IL-8 or CXCR2 inhibitors—to target both angiogenic and inflammatory pathways simultaneously [49,50].

Despite these promising insights, the study has several limitations. Due to patient mortality and logistical constraints, matched pre- and post-chemotherapy samples were not obtained in all cases, limiting the power of longitudinal comparisons. As a limitation of this research, none of the patients received a VEGF-targeted agent. Therefore, predictive biomarker utility could not be evaluated. The baseline data showed no significant prognostic association between tissue *VEGF-A* expression and survival; however, we cannot show that *VEGF-A* expression identifies patients who will respond to bevacizumab, ramucirumab, or another anti-angiogenic agent. For further research, we may design research methods in relation to specific targeted therapies more effectively based on these results in our gastric cancer patient population. Moreover, molecular features such as microsatellite instability, EBV status, and HER2 amplification were not assessed in this study; these could provide more clinical data and an additional biological profile of *VEGF-A* and *IL-8* regulation in advanced gastric cancer patients. Finally, despite complete follow-up data regarding overall survival outcomes, the overall sample size remains modest, necessitating an exploratory interpretation of the multivariable Cox regression estimates.

Nonetheless, this study contributes novel data from a previously underrepresented Thai population and underscores the importance of molecular profiling in gastric cancer. Overall, our findings indicate that baseline expression of *VEGF-A* and *IL-8* does not directly predict survival, but post-chemotherapy *VEGF-A* induction highlights a potential individualization for precision medicine that merits further investigation. However, this important profile may allow us to explore further in relation to targeted therapy. Given the limited sample size and observational nature of our cohort, these observations remain strictly exploratory, and future prospective trials involving anti-angiogenic regimens are warranted to determine their clinical applicability.

## 5. Conclusions

In conclusion, this study provides an exploratory overview of *VEGF-A* and *IL-8* expression profiles in a Northern Thai gastric cancer cohort. Rather than establishing these molecules as definitive prognostic biomarkers, our baseline findings reveal marked inflammatory gene expression heterogeneity, with *IL-8* upregulation occurring more frequently than *VEGF-A* and VEGF-A gene expression being less frequent. Importantly, longitudinal analysis demonstrated that standard chemotherapy failed to consistently suppress *VEGF-A* expression; in fact, post-treatment *VEGF-A* induction was observed in a subset of patients, whereas *IL-8* levels exhibited dynamic fluctuations. These observations suggest that post-chemotherapy *VEGF-A* reactivation might represent a potential therapeutic vulnerability. Patients exhibiting such *VEGF-A* surges may warrant further investigation to explore whether combining targeted anti-VEGF-A agents with conventional chemotherapy could prove beneficial. Nevertheless, due to the small sample size and observational nature of this subgroup analysis, these findings remain hypothesis-generating and underscore the need for larger prospective cohorts to evaluate the relevance of further targeted therapy and monitoring longitudinal expression profiles in gastric cancer treatment in future research.

## Figures and Tables

**Table 1:** Clinical characteristics of gastric cancer patients.

Group		IL-8		VEGF-A	
		Pre-Chemotherapy (*n* = 52) n (%)	Post-Chemotherapy (*n* = 8) n (%)	Pre-Chemotherapy (*n* = 74) n (%)	Post-Chemotherapy (*n* = 14) n (%)
Sex	Female	14 (26.92)	3 (37.50)	26 (35.13)	5 (35.71)
	Male	22 (42.31)	3 (37.50)	28 (37.84)	5 (35.71)
	N/A	16 (30.77)	2 (25.00)	20 (27.03)	4 (28.58)
Age	<55	18 (34.62)	2 (25.00)	23 (31.08)	5 (35.71)
	≥55	24 (46.15)	4 (50.00)	38 (51.35)	5 (35.71)
	N/A	10 (19.23)	2 (25.00)	13 (17.57)	4 (28.58)
Histological grade	Well differentiated	6 (11.54)	3 (37.50)	12 (16.22)	4 (28.57)
	Moderately differentiated	5 (9.62)	1 (12.50)	5 (6.76)	2 (14.28)
	Poorly differentiated	33 (63.46)	3 (37.50)	46 (62.16)	6 (42.86)
	N/A	8 (15.38)	1 (12.50)	11 (14.86)	2 (14.29)
Surgical treatment	Palliative surgery	10 (19.24)	1 (12.50)	16 (21.62)	3 (21.43)
	Definitive surgery (gastrectomy)	21 (40.38)	4 (50.00)	31 (41.89)	5 (35.71)
	Palliative care	21 (40.38)	3 (37.50)	27 (36.49)	6 (42.86)
Adjuvant or Neoadjuvant	Adjuvant	19 (36.54)	0 (0.00)	28 (37.84)	9 (64.29)
	Neoadjuvant	33 (63.46)	8 (100.00)	22 (29.73)	5 (35.71)
	Palliative chemoRx.	0 (0.00)	0 (0.00)	1 (1.35)	0 (0.00)
	N/A	0 (0.00)	0 (0.00)	23 (31.08)	0 (0.00)
Stage	I	1 (1.92)	0 (0.00)	1 (1.35)	0 (0.00)
	II	5 (9.62)	2 (25.00)	7 (9.46)	1 (7.14)
	III	4 (7.69)	0 (0.00)	10 (13.51)	0 (0.00)
	IV	24 (46.15)	4 (50.00)	33 (44.60)	8 (57.15)
	N/A	18 (34.62)	2 (25.00)	23 (31.08)	5 (35.71)

**Table 2:** Multivariate cox regression analysis and survival rate of *IL-8* and *VEGF-A* expression profile (*n* = 15).

	Overall n (%)	Mean ± SD (Months)	Median Survival (Months)	Univariable Analysis			Multivariable Analysis		
Characteristic	*n* = 15 ^1^	*n* = 15 ^2^	50% Percentile	HR	95% CI	*p*-Value	HR	95% CI	*p*-Value
Sex									
Male	10 (67.67%)	29.28 ± 15.20	28	—	—		—	—	
Female	5 (33.33%)	16.32 ± 14.31	12	2.39	0.76, 7.54	0.14	3.31	0.47, 23.2	0.23
Age									
<55	5 (33.33%)	29.88 ± 20.83	27	—	—		—	—	
≥55	10 (67.67%)	22.50 ± 13.09	18	1.77	0.54, 5.78	0.34	8.97	1.07, 75.3	0.043
Histologic grade									
Well-differentiated	3 (20.00%)	16.16 ± 13.80	15	—	—		—	—	
Moderately differentiated	2 (13.33%)	21.09 ± 12.64	21	0.85	0.14, 5.21	0.86	0.49	0.05, 4.83	0.54
Poorly differentiated	10 (67.67%)	28.37 ± 16.80	24	0.42	0.10, 1.70	0.22	1.14	0.19, 6.78	0.88
Treatment type									
Neoadjuvant	7 (46.67%)	20.10 ± 11.68	16	—	—		—	—	
Adjuvant	8 (53.33%)	29.21 ± 18.24	29	0.49	0.16, 1.50	0.21	0.10	0.01, 0.77	0.027
IL-8 Expression									
Normal expression	1 (6.67%)	3.22 ± NA	3.2	—	—		—	—	
Overexpression	14 (93.33%)	26.51 ± 15.07	24	0.00	0.00, Inf	>0.99	0.00	0.00, Inf	>0.99
VEGF Expression									
Normal expression	10 (66.67%)	22.08 ± 16.49	18	—	—		—	—	
Overexpression	5 (33.33%)	30.71 ± 13.80	30	0.69	0.23, 2.08	0.51	0.19	0.04, 0.97	0.046

Note: *n* = 15 for the multivariable Cox regression analysis and the survival analysis of *IL-8* expression profiles, with 15 recorded deaths (0% censored). Expression levels of *IL-8* and *VEGF-A* were determined relative to matched normal mucosal tissues (Log_10_ fold change, with baseline relative to AGS reference cells); “Normal expression” was defined as Log_10_ fold change < 1, and “Overexpression” was defined as Log_10_ fold change ≥ 1. ^1^ n (%); ^2^ time_months: Mean ± SD; Abbreviations: CI = Confidence Interval, HR = Hazard Ratio.

## Data Availability

The data presented in this study are available on request from the corresponding author due to ethical reasons.

## Abbreviations

The following abbreviations are used in this manuscript:

GC	Gastric cancer
IL-8	Interleukin-8
VEGF-A	Vascular endothelial growth factor A
